# Retinal and choroidal vascular abnormalities in TTR-FAP

**DOI:** 10.1186/1750-1172-10-S1-P61

**Published:** 2015-11-02

**Authors:** Antoine Rousseau, Emmanuel Barreau, Céline Terrada, Lydie Bonin, Pierre-Raphaêl Rothschild, Marie Théaudin, David Adams, Marc Labetoulle

**Affiliations:** 1 French Reference Center for FAP (NNERF), Bicêtre Hospital, Ophthalmology, 94275, Le Kremlin-Bicêtre, France; 2 Reference Centre for Rare Vascular Diseases of the Central Nervous System and the Retina, Lariboisière Hospital, Ophthalmology, 75010, Paris, France; 3 Cochin Hospital, Ophthalmology, 75014, Paris, France; 4 French Reference Center for FAP (NNERF), Bicêtre Hospital, Neurology, 94275, Le Kremlin-Bicêtre, France

## Background

Retinal amyloid angiopathy is considered to be a rare ocular manifestation of TTR-FAP. Choroidal vascular abnormalities have been described in isolated case reports. The purpose of this study was to describe fluorescein and indocyanine green angiographic findings of retinal and choroidal vascular abnormalities in a series of TTR-FAP patients.

## Methods

This monocentric observational study was conducted at the French National Reference Center for TTR-FAP. Genetically confirmed TTR-FAP patients with suspected retinal and/or choroidal vascular abnormalities underwent fluorescein and indocyanine green angiography. Sensorimotor polyneuropathy (SPN) was staged with the Polyneuropathy Disability (PND) score, vegetative neuropathy was staged with the Compound Autonomic Dysfunction Test (CADT). Medical and surgical treatments were analyzed for all patients.

## Results

Twelve patients (8 males and 4 females), aged 47-82 years (mean 55.8±14.1 years) were included. Mean delay between first symptoms and inclusion was 9.6±5.3 years. Val30Met mutation was present in 10/12 patients. Bilateral retinal ischemic vasculopathy was present in 8/12 patients and included retinal hemorrhages, microaneurysms, venous and arteriolar segmental staining. Retinal ischemia lead to preretinal neovascularization in one patient and to neovascular glaucoma in 5 eyes of 3 patients. Treatments included panretinal photocoagulation (10 eyes, 5 patients), intravitreal anti-VEGF injections (4 eyes, 2 patients) and trabeculectomy (both eyes of one patient). Vitreous amyloid deposits were present in 7/8 cases of retinal amyloid angiopathy. Typical bilateral amyloid choroidal vasculopathy was found in 9/12 patients. It consisted in late and diffuse staining of the arterial choroidal vasculature, and was associated with vitreous amyloid deposits in 7/9 cases. Anterior chamber amyloid deposits were present in 2/8 patients with retinal amyloid angiopathy and in 3/9 patients with choroidal amyloid angiopathy. All the studied patients had a PND score ≥ 1 and 10/12 patients had dysautonomic symptoms.

## Conclusions

Retinal amyloid angiopathy can be considered as a severe form of ischemic retinopathy. Clinical significance of choroidal amyloid angiopathy remains unknown. Further studies are warranted to determine the potential systemic counterparts of ocular amyloid angiopathies.

